# Transcranial Doppler ultrasound in vascular cognitive impairment-no dementia

**DOI:** 10.1371/journal.pone.0216162

**Published:** 2019-04-24

**Authors:** Luisa Vinciguerra, Giuseppe Lanza, Valentina Puglisi, Manuela Pennisi, Mariagiovanna Cantone, Alessia Bramanti, Giovanni Pennisi, Rita Bella

**Affiliations:** 1 Neurology Department and Stroke Unit, IRCCS Centro Neurolesi “Bonino Pulejo”, Messina, Italy; 2 Department of Surgery and Medical-Surgical Specialties, University of Catania, Catania, Italy; 3 Department of Neurology IC, Oasi Research Institute – IRCCS, Troina, Italy; 4 Department of Biomedical and Biotechnological Sciences, University of Catania, Catania, Italy; 5 Neurology Department, “Sant’Elia” Hospital, ASP Caltanissetta, Caltanissetta, Italy; 6 IRCCS Centro Neurolesi “Bonino Pulejo”, Institute of Applied Sciences and Intelligent Systems (ISASI), Messina, Italy; 7 Department of Medical and Surgical Sciences and Advanced Technology, Section of Neurosciences, University of Catania, Catania, Italy; Nathan S. Kline Institute, UNITED STATES

## Abstract

**Background:**

Although cerebral white matter lesions (WMLs) are considered as a risk factor for vascular dementia, data on their impact on cerebral hemodynamics are scarce. We test and compare transcranial Doppler (TCD) features in WML patients with or without associated cognitive impairment.

**Methods:**

A sample of non-demented elderly patients with WMLs was consecutively recruited. Mean blood flow velocity (MBFV), pulsatility index (PI), peak systolic blood flow velocity (PSV), end-diastolic blood flow velocity (EDV), and resistivity index (RI) were recorded from the middle cerebral artery bilaterally. Global cognitive functioning, frontal lobe abilities, functional status, and WML severity were also assessed.

**Results:**

161 patients satisfying the clinical criteria for vascular cognitive impairment-no dementia (VCI-ND) were age-matched with 97 presenting WMLs without any cognitive deficit. VCI-ND patients exhibited a decrease in MBFV and EDV, as well as an increase in PI, RI, and PSV. Moreover, a significant correlation between all TCD parameters and the severity of executive dysfunction was observed, whereas PI, RI, and EDV were significantly correlated with the WML load.

**Conclusions:**

VCI-ND showed a hemodynamic pattern indicative of cerebral hypoperfusion and enhanced vascular resistance. These changes may be considered as the TCD correlate of VCI-ND due to microcirculation pathology. TCD provides useful indices of the occurrence and severity of small vessel disease and executive dysfunction in elderly patients at risk of future dementia.

## Introduction

Vascular cognitive impairment (VCI) defines the wide spectrum of cognitive disorders caused by different types of cerebrovascular disease. Within this frame, the term VCI-no dementia (VCI-ND) describes those subjects whose cognitive decline is not associated with substantial functional impairment. Although VCI-ND is not necessarily predictive of a progression into overt dementia [1], it represents a higher risk to develop more severe forms of cognitive impairment either as major VCI or mixed degenerative and vascular dementia (VaD), especially after recurrent strokes [2].

Growing evidence correlates cerebral hypoperfusion to both cognitive decline and white matter lesions (WMLs) [3]. Previous studies have applied transcranial Doppler ultrasound (TCD) to explore the relationship between cerebral hemodynamics and brain lesions attributed to small vessel disease in cognitive disorders [3–6]. The rationale is that microangiopathy, demonstrated in both vascular and degenerative dementia, might lead to arteriolosclerotic processes, vasoconstriction, and vascular stiffness, thus resulting in decreased arterial diameter and cerebral blood flow [3,7,8]. In this scenario, TCD is a well-known and feasible tool to evaluate the cerebral hemodynamics, the arterial perfusion integrity, and the intracranial small vessel compliance [9–11].

Although TCD has been employed in patients with VaD [3,7,8,12,13], no comparable data are available in those with VCI-ND. The aim of this study was to assess indices of cerebral blood flow velocity in VCI-ND patients and to correlate TCD changes with neuropsychological scores and WML severity. We hypothesized that alterations in cerebral hemodynamic may affect cognition even in the absence of overt dementia.

## Methods

### Subjects

This was a cross-sectional study that consecutively recruited all elderly patients (65 years or older) satisfying the clinical criteria for VCI-ND [14] and those with WMLs without any cognitive deficit. All subjects attended the Cerebrovascular Disease outpatient Clinic of the University Hospital of Catania (Italy) between December 2016 and April 2018. This center is dedicated to the diagnosis, treatment, and follow-up of patients with transient ischemic attack (TIA) and post-acute stroke, patients with chronic subcortical cerebrovascular disease without any history of stroke, and patients with vascular-related cognitive impairment (from mild [VCI-ND] to severe forms [VaD and its subtypes]). According to the latest Classification Consensus Study of the Vascular Impairment of Cognition [1], VCI-ND identifies a clinically and radiologically homogeneous group of non-demented patients affected by small vessel disease (lacunar infarcts and ischemic white matter lesions) primarily located subcortically.

The diagnosis of VCI-ND was based on the proposed criteria of the International Society for Vascular Behavioral and Cognitive Disorders [15]. According to these criteria, patients were included in this study if they had evidence of cognitive decline in specific domains (speed of information processing, complex attention, frontal-executive functioning) and significant neuroimaging (magnetic resonance imaging [MRI] or computed tomography) abnormalities indicative of a cerebrovascular disease, in the absence of autonomy loss and with no history or radiological evidence of stroke. Therefore, the inclusion criteria were based on medical history, neurological examination, psychocognitive assessment, and neuroradiological investigation. VCI-ND patients showed impairment in at least one cognitive domain and normal functional independence in the activities of daily living [1,2,15]. None of the participants met the Diagnostic and Statistical Manual for Mental Disorders—Fifth Edition (DSM-V) criteria for dementia [16], whereas they all fulfilled the MRI criteria for subcortical ischemic vascular disease with predominant WMLs [17].

Exclusion criteria were: Mini-Mental State Examination (MMSE) score ≤ 24 [18]; history of stroke/TIA or other neurological disorders (e.g., Parkinson’s disease, head trauma, epilepsy); major psychiatric disorders (e.g., schizophrenia, obsessive-compulsive disorder, depressive disorders); any uncompensated acute or chronic medical illness; endocrinopathies affecting cognitive functions; alcohol or drug abuse; ultrasound evidence of carotid or vertebral extracranial artery stenosis ≥ 50% prior to the enrolment; ultrasound evidence of intracranial artery stenosis; bilateral absence of adequate transtemporal windows for TCD examination; any condition precluding MRI.

This study was carried out in accordance with the Declaration of Helsinki of 1964 and its later amendments. The protocol was approved by the Ethics Committee of the “Azienda Ospedaliero-UniversitariaPoliclinico-Vittorio Emanuele” of Catania (Italy). All subjects gave written informed consent prior to study entry.

### Assessment

The clinical and demographic assessment included: age, sex, education, presence of cardio- and cerebrovascular risk factors, and neurological signs and symptoms. All patients were treated for their vascular risk factors with antiplatelet or anticoagulant medications (aspirin, clopidogrel, warfarin), antihypertensive drugs (angiotensin-converting enzyme inhibitors, angiotensin II receptor antagonist, diuretics, calcium channel blockers if necessary), cholesterol-lowering medications (statins), and oral antidiabetic drugs or insulin.

All participants underwent a neuropsychological battery of tests, including: global screening tools of cognitive impairment (MMSE and Montreal Cognitive Assessment Test [MoCA]) [19], evaluation of the functional status by basic and instrumental activities of daily living (Activity of Daily Living [ADL] and Instrumental Activity of Daily Living [IADL]), and evaluation of executive functions using the Stroop Color-Word Test interference (normative values collected from an Italian population sample, Stroop T score ≤ 36.92 s [20]). Briefly, the Stroop test is a measure of attention and response inhibition to conflicting stimuli and explores the functional integrity of the dorsolateral prefrontal cortex and anterior cingulate cortex engaged during the task [21]. Practically, it consists of three tables with the names of colors, the color patches, and the names of color printed in a conflicting color ink (e.g., the word “red” in green ink) that the subjects have to read. In the third task, it is required to name the color of the ink rather than the word, so that participants have to inhibit the automated task of reading the word, naming the ink color [22]. As such, Stroop T is also in VCI-ND patients widely considered as a highly sensitive and reliable tool to evaluate executive functioning, cognitive flexibility, and control performances, as demonstrated in a recent systematic review and meta-analysis [23].

Brain MRIs were acquired using a 1.5-T General Electric system. The protocol included T1-, T2-, proton density-weighted, and fluid-attenuated inversion recovery scans; the slice thickness was 5 mm with a 0.5 mm slice gap. The severity of deep WMLs was graded according to the visual scale of Fazekas: 0 = absence; 1 = punctuate foci; 2 = partially confluent foci; 3 = large confluent areas [24].

### TCD protocol

TCD sonography (Compumedics DWL, Multi-Dop X digital, Singen, Germany) was performed by a trained sonographer (RB) who was blinded to the patient’s neuropsychological performance and WML load. The blood flow velocity of the proximal tract (M1) of the middle cerebral artery was bilaterally recorded with a handheld 2-MHz DWL Ultrasound Probes PW through the temporal bone window under resting conditions and at the depth that provided the best signal (50–60 mm).

The following parameters were evaluated: peak systolic blood flow velocity (PSV), end-diastolic blood flow velocity (EDV), mean blood flow velocity (MBFV), pulsatility index (PI) calculated according to the formula (PSV-EDV)/MBFV by Gosling and King [25], and the Pourcelot resistivity index (RI) that was equal to (PSV-EDV)/PSV.

TCD values were acquired after a 30-s stable recording period and lasted at least for 10 cardiac cycles [26]. The parameters were obtained as the mean of two measurements on each side; otherwise, the measurements from the only available side were used. Data were stored on a PC for off-line analysis. Heart rate and mean arterial pressure were recorded at the time of the examination.

### Statistical analysis

Statistical software (SPSS, version 22.0) was used for all analyses. Data were reported as number and percentage, mean ± standard deviation, or the median and interquartile range, as appropriate. Patient’s characteristics and TCD parameters (MBFV, PSV, EDV, PI, RI) were compared by using the Mann-Whitney test for skewed continuous data and the chi-square test for categorical variables. The correlation analysis between TCD parameters and severity of WMLs, cognitive functions, and executive dysfunction was performed by means of Spearman’s correlation coefficients. The associations between TCD parameters and WML loads or performances on cognitive tests in VCI-ND patients were assessed with linear regression models, adjusted for age, sex, education, and vascular risk factors. For all analyses, a *p*-value < 0.05 was considered a statistically significant difference.

## Results

From a total sample of 413 participants that were originally screened, 103 were excluded due to an MMSE ≤ 24 or a loss of autonomy according to the ADL and/or IADL scores, 25 presented with insufficient/absent acoustic transtemporal windows bilaterally, MRI was contraindicated in 21 patients due to different reasons (metallic implants, pacemakers and implantable defibrillators, claustrophobia, etc.), and 6 refused to participate. Therefore, a total of 258 subjects satisfying the study criteria were finally enrolled and classified into two groups based on the results of the neuropsychological tests: 161 VCI-ND patients (Group A) and 97 patients with WMLs without any cognitive deficit (Group B). Demographic features, vascular risk factors, neuropsychological scores, neuroradiological findings, and TCD measures are reported in Table 1.

**Table 1:** Clinical-demographic characteristics, neuroradiological features, neuropsychological test scores, and TCD values of all participants.

Variable	Group B (n = 97)	Group A (n = 161)	*p*
Age, mean ± SD (years)	68.58 ± 6.04	73.63 ± 7.07	**<0.001**
Sex Male (%) Female (%)	47 (48.5) 50 (51.5)	87 (54.0) 74 (46.0)	0.45
Educational level, mean ± SD (years)	8.34 ± 4.25	6.86 ± 3.67	**0.006**
Smokers (%) Ex-smokers (%)	23 (23.7) 14 (14.4)	31 (19.3) 19 (18.8)	0.51
Hypertension (%)	77 (79.4)	130 (80.7)	0.80
Hypercholesterolemia (%)	29 (29.9)	58 (36.0)	0.31
Diabetes (%)	24 (24.7)	48 (29.8)	0.38
Coronaropathy (%)	9 (9.3)	30 (18.6)	**0.042**
Atrial fibrillation (%)	7 (7.2)	22 (13.7)	0.11
MMSE, median (I-III quartile)	27.00 (26.00–29.00)	25.0 (24.00–26.00)	**<0.001**
Stroop T score (I-III quartile)	24.50 (16.90–30.85)	50.3 (41.00–71.00)	**<0.001**
MoCA (I-III quartile)	24.00 (23.00–27.00)	21.0 (20.00–23.00)	**<0.001**
MRI Fazekas visual scale Grade 1 (%) Grade 2 (%) Grade 3 (%)	55 (56.7) 37 (38.1) 5 (5.2)	40 (24.8) 69 (42.9) 52 (32.3)	**<0.001**
MBFV, median (I-III quartile)	59.00 (57.00–60.00)	54.00 (51.00–55.00)	**<0.001**
PI, median (I-III quartile)	0.70 (0.59–0.80)	0.95 (0.83–1.08)	**<0.001**
RI, median (I-III quartile)	0.48 (0.42–0.52)	0.59 (0.53–0.62)	**<0.001**
PSV, median (I-III quartile)	85.10 (81.70–89.44)	86.20 (82.56–90.93)	0.31
EDV, median (I-III quartile)	45.00 (42.25–47.37)	36.80 (33.18–39.42)	**<0.001**

SD: standard deviation; MMSE: Mini-Mental State Examination; MoCA: Montreal Cognitive Assessment; MRI: magnetic resonance imaging; MBFV: mean blood flow velocity; PI: pulsatility index; RI: resistivity index; PSV: peak systolic blood flow velocity; EDV: end-diastolic blood flow velocity; bold numbers: statistically significant *p*-values.

None of the participants had focal motor deficits; slight reflex asymmetry was present in 16 patients of Group A and 9 of Group B, whereas unsteadiness was detected in 6 and 2 patients, respectively. Group A patients were older (*p*< 0.001) and less educated than Group B (*p* = 0.006), whereas sex did not differ between these groups. No difference in vascular risk factors was found, except for coronaropathy that was more prevalent in Group A (*p* = 0.042). In addition, Group A patients showed more severe WMLs than Group B (*p*< 0.01).

The mean insonation depth of the M1 segment of the middle cerebral artery was 55 ± 5 mm. An adequate transtemporal window for TCD measurements was achieved in 90.3% of the study subjects. Compared to Group B, Group A exhibited lower values for MBFV and EDV (*p*< 0.01), but higher values for PI and RI (*p*< 0.01; see S1 Fig).

The Spearman correlation detected a significant association between all TCD parameters and Stroop T. In particular, PI, RI, and PSV were directly correlated to Stroop T (*p*< 0.01), whereas PI and RI were inversely correlated to MMSE (*p<* 0.05) and MoCA (*p<* 0.01). The values for MBFV and EDV were inversely correlated to Stroop T values (*p<* 0.01), whereas the EDV was directly correlated to the scores in the MoCA (*p<* 0.01) and the MMSE (*p* = 0.04; see S2 Fig).

Table 2 shows the regression models in Group A. PI was significantly and independently associated with Stroop T (*p*< 0.001) and WML severity (*p*< 0.001). Similarly, PI (*p*< 0.001), as well as RI (*p*< 0.001) and EDV (*p* = 0.001) were associated with Stroop T and WML severity, whereas PSV was associated with Stroop T (*p* = 0.028). A reduction in MBFV did not predict cognitive dysfunction or WML severity. The multivariate linear regression models performed in Group B did not produce any significant result. The analysis adjusted for age, education, and vascular risk factors did not show any influence of these factors on the aforementioned results. Moreover, even when regression has included the whole sample of subjects (Group A + B), the results did not differ in terms of lack of significant effect of age. This variable, indeed, did not reach the statistical significance needed as a confounding factor.

**Table 2:** Backward linear regression: Significant predictors of TCD parameters in VCI-ND patients (Group A).

Dependent variables	Predictors	Std β	*p*	Adjusted R^2^
PI	WMLs	0.30	**<0.001**	0.33
	Stroop T	0.40	**<0.001**	
MBFV	WMLs	-0.16	0.063	0.002
	Stroop T	-0.05	0.59	
RI	WMLs	0.30	**<0.001**	0.25
	Stroop T	0.32	**<0.001**	
EDV	WMLs	-0.30	**0.001**	0.19
	Stroop T	-0.30	**<0.001**	
PSV	WMLs	0.46	0.60	0.02
	Stroop T	0.20	**0.028**	

PI: pulsatility index; MBFV: mean blood flow velocity; RI: resistivity index; EDV: end-diastolic blood flow velocity; PSV: peak systolic blood flow velocity; WMLs: white matter lesions; Stroop T: Stroop Color-Word Test interference; bold numbers: statistically significant *p*-values.

## Discussion

To the best of our knowledge, this is the first TCD study investigating cerebral hemodynamics in VCI-ND patients. While different studies investigating cerebral hemodynamics in Mild Cognitive Impairment (MCI) of degenerative cause have been published, no study evaluating TCD parameters in a large cohort of non-demented patients with cognitive decline of vascular origin (VCI-ND) has been available until now.

The main finding of this study is that specific measures of cerebral perfusion and vascular resistance were significantly associated with WMLs and executive performance in patients with VCI-ND. It is worth to highlight that the literature provides limited data on the hemodynamic changes in the preclinical stages of dementias. In particular, previous reports failed to demonstrate PI or MBFV differences between patients with MCI and healthy controls [27,28]. Conversely, PI was increased in both VaD [8,29] and Alzheimer’s disease (AD) with respect to healthy controls [12,27,29], although some reports found a higher PI in VaD than in AD [13,30].

In the present study, although the MBFV was lower in VCI-ND patients than in those without, it was not independently predictive of cognitive impairment, possibly reflecting only a partially altered cerebral hemodynamics at this stage. A reduced MBFV might be due to the hypoperfusion secondary to arterial sclerosis [31]. Compared to PI, however, MBFV seems to be a weaker TCD index for impaired cerebral hemodynamics in cognitively impaired subjects. Indeed, previous studies did not show a significant difference in MBFV between demented patients and controls [30,32]. Nevertheless, a recent meta-analysis concluded that MBFV is lower in both AD and VaD patients compared to controls, although disturbances in cerebral hemodynamics were more severe in VaD, suggesting a specific spectrum of hemodynamic changes in different types of dementia [7].

Overall, the TCD changes detected in VCI-ND revealed a global pattern of cerebral hypoperfusion and increased vascular resistance, likely due to microcirculation pathology and linked to small vessel and capillary damage. These findings have been extensively demonstrated in VaD [29] but also in AD [33] and are primarily related to lipohyalinosis processes, supporting the role of vascular contribution in neurodegeneration. Furthermore, the increase of PSV, RI, and PI that we observed in VCI-ND patients can be considered as the correlate of distal arterial constriction or increased downstream resistance. Indeed, the increase in PI indirectly indicates a rise of vascular peripheral resistances and might also be explained by the diastolic reduction in flow velocities and the cerebral vascular compliance decline [3,7,8]. Therefore, the combination of high vascular resistances and low perfusion state suggests a global vascular impairment, possibly starting from small vessels and then spreading to the larger arteries. In this regard, some authors proposed that PI represents a marker of small vessel disease [34]. Interestingly, the severity of hemodynamic alterations detected by TCD might also reflect the severity of WMLs. Accordingly, we found that increased PI values resulted independently from other factors in more extensive WMLs on brain MRI [5,34,35]. Similarly, PI was a predictor of both worse cognitive performance and brain lesion load.

The results of this study should also consider that the subcortical white matter is markedly vulnerable to hypoperfusion and subsequent ischemia because of poorly vascularization by perforating arteries [36]. Hypoperfusion might cause ischemic injury of white matter tracts and interruption of the subcortical circuits, thus resulting in various cognitive symptoms and, particularly, in executive dysfunction [37,38]. Hence, TCD flow pattern of the large arteries stiffness is also associated with WML burden.

A strength of the present study is the enrolment of a large and representative sample of patients with VCI-ND caused by subcortical ischemic vascular disease. Subjects were also stratified according to their WML load, which is a marker of small vessel disease progression. Additionally, the TCD findings observed may contribute to the further understanding of the process and progress of VCI and represent an additional tool to disentangle the mixed forms of cognitive decline, even in the early stages. Lastly, VaD can be prevented. Therefore, TCD may be added as a clinical routine examination to detect patients who can benefit from an intensive medical therapy. One of the limitations is the lack of healthy controls, although the difficulties in recruiting a comparable number of age-matched healthy subjects without any imaging evidence of subcortical ischemic vascular disease (commonly observed among elderly) and cognitive impairment in neuropsychological tests should be taken into account. Moreover, the recruitment was conducted in a single center, and no follow-up was carried out.

## Conclusion

TCD provides useful indices of correlation between WMLs and executive dysfunction in VCI-ND. Since the cerebral hypoperfusion is regarded as both a risk factor and an aggravating component of cognitive decline in the elderly, TCD represents a valuable tool to screen subjects at risk of both vascular and mixed dementia. Future studies are needed to acquire a more comprehensive understanding of the complex relationships between hemodynamic changes, WMLs, and cognitive decline. The possibility to early detect any marker of transition into overt dementia will support clinicians towards a more careful diagnosis and management of this population at risk.

## Supporting information

S1 Fig Box plot of TCD parameters in Group A and Group B. TCD, transcranial Doppler ultrasonography; PI, pulsatility index; MBFV, mean blood flow velocity; RI, resistivity index; EDV, end-diastolic blood flow velocity.
(PDF)

S2 Fig Correlation between TCD parameters and cognitive tests. PI, pulsatility index; MBFV, mean blood flow velocity; RI, resistivity index; PSV, peak systolic blood flow velocity; EDV, end-diastolic blood flow velocity; MMSE, Mini-Mental State Examination; MoCA, Montreal Cognitive Assessment Test; Stroop T, Stroop Color—Word Test interference.
(PDF)

## Abbreviations

AD: Alzheimer’s disease
ADL: Activity of Daily Living
EDV: end-diastolic blood flow velocity
IADL: Instrumental Activity of Daily Living
MBFV: mean blood flow velocity
MCI: Mild Cognitive Impairment
MMSE: Mini-Mental State Examination
MoCA: Montreal Cognitive Assessment Test
MRI: magnetic resonance imaging
PI: pulsatility index
PSV: peak systolic blood flow velocity
RI: resistivity index
Stroop T: Stroop Color—Word Test interference
TCD: transcranial Doppler
TIA: Transient Ischemic Attack
VCI: vascular cognitive impairment
VCI-ND: vascular cognitive impairment-no dementia
WMLs: white matter lesions
