# Supplementary figures and images for: Transcranial Doppler ultrasound in vascular cognitive impairment-no dementia
Source: PLoS One. 2019 Apr 24;14(4):e0216162. doi: 10.1371/journal.pone.0216162 (PMC6481922; doi:10.1371/journal.pone.0216162)

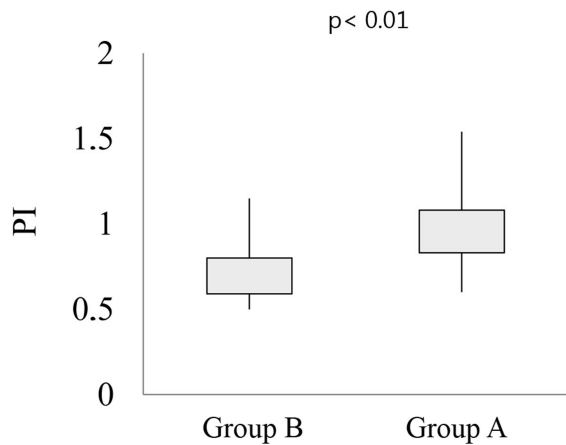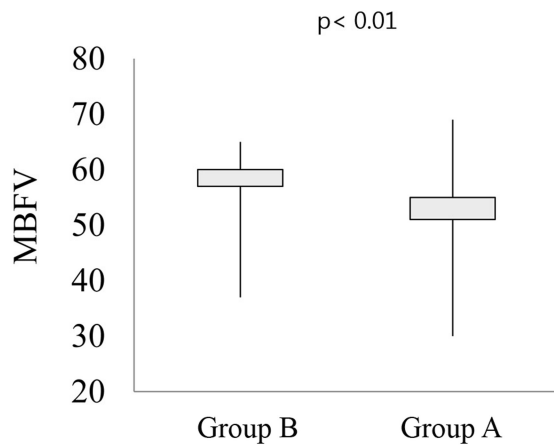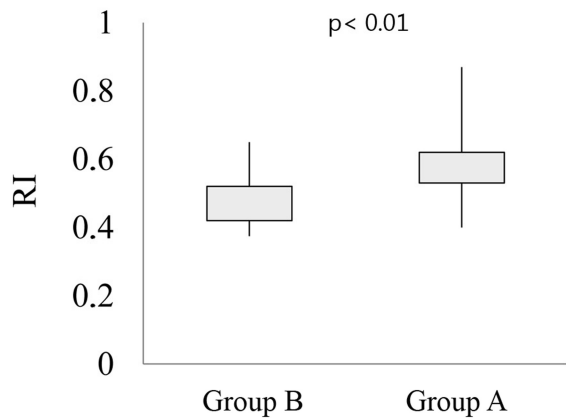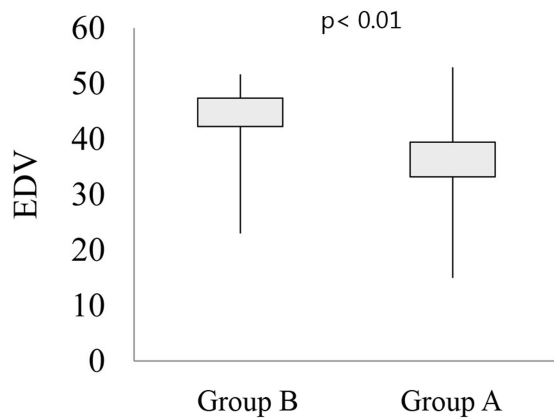

Supplement: S1 Fig — TCD, transcranial Doppler ultrasonography; PI, pulsatility index; MBFV, mean blood flow velocity; RI, resistivity index; EDV, end-diastolic blood flow velocity. (PDF) [file pone.0216162.s001.PDF]

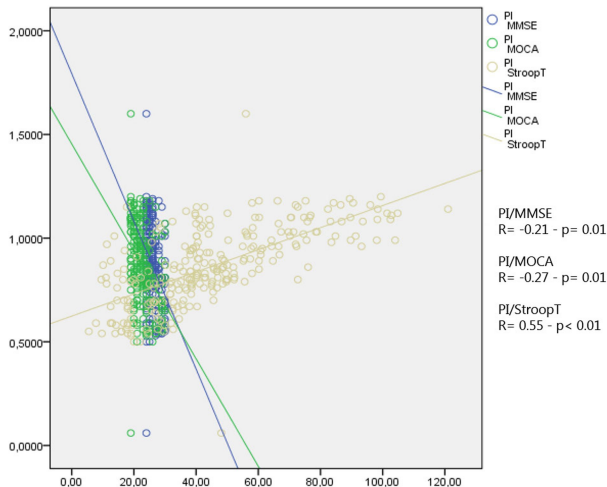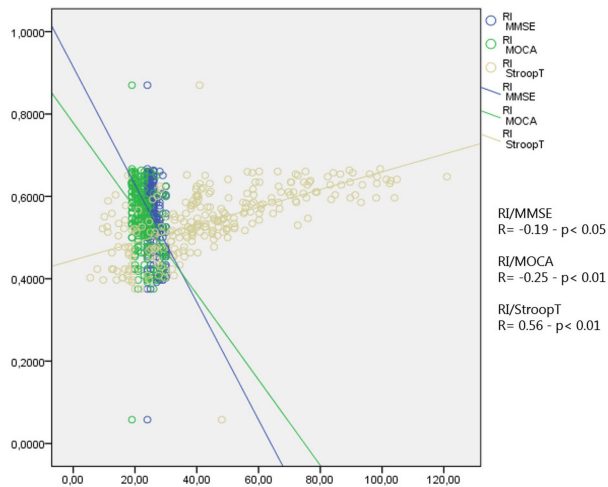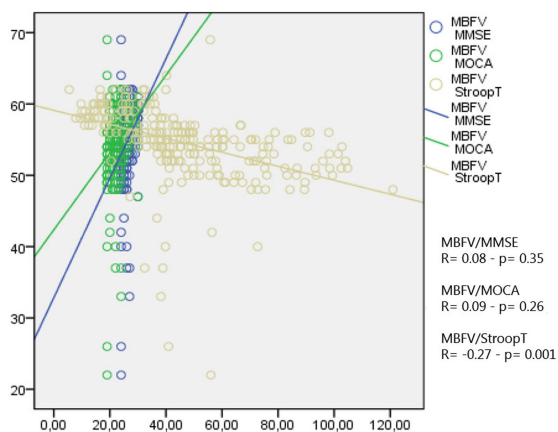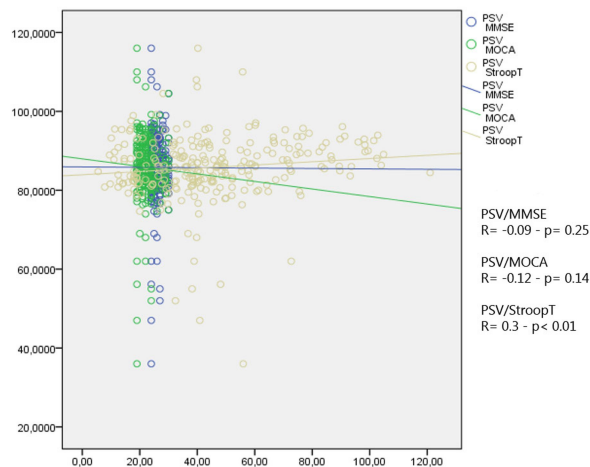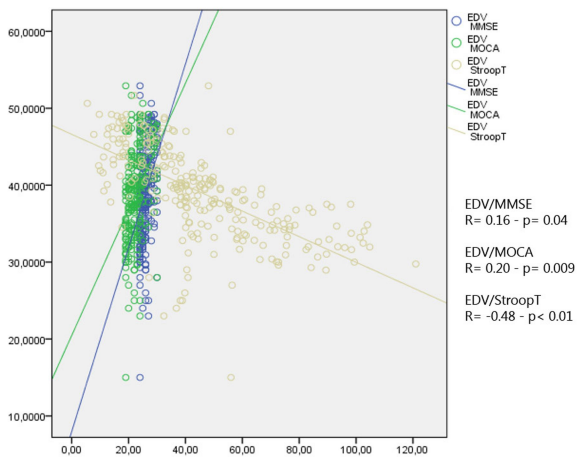

Supplement: S2 Fig — PI, pulsatility index; MBFV, mean blood flow velocity; RI, resistivity index; PSV, peak systolic blood flow velocity; EDV, end-diastolic blood flow velocity; MMSE, Mini-Mental State Examination; MoCA, Montreal Cognitive Assessment Test; Stroop T, Stroop Color—Word Test interference. (PDF) [file pone.0216162.s002.pdf]
